# Supplementary material for: Hepatic arginase 2 (Arg2) is sufficient to convey the therapeutic metabolic effects of fasting
Source: Nat Commun. 2019 Apr 8;10:1587. doi: 10.1038/s41467-019-09642-8 (PMC6453920; doi:10.1038/s41467-019-09642-8)
Supplement: Supplementary file 1 — Supplementary Information [file 41467_2019_9642_MOESM1_ESM.pdf]

Supplementary Information

Hepatic Arginase 2 (Arg2) is sufficient to convey the therapeutic metabolic effects of fasting

Zhang et al.

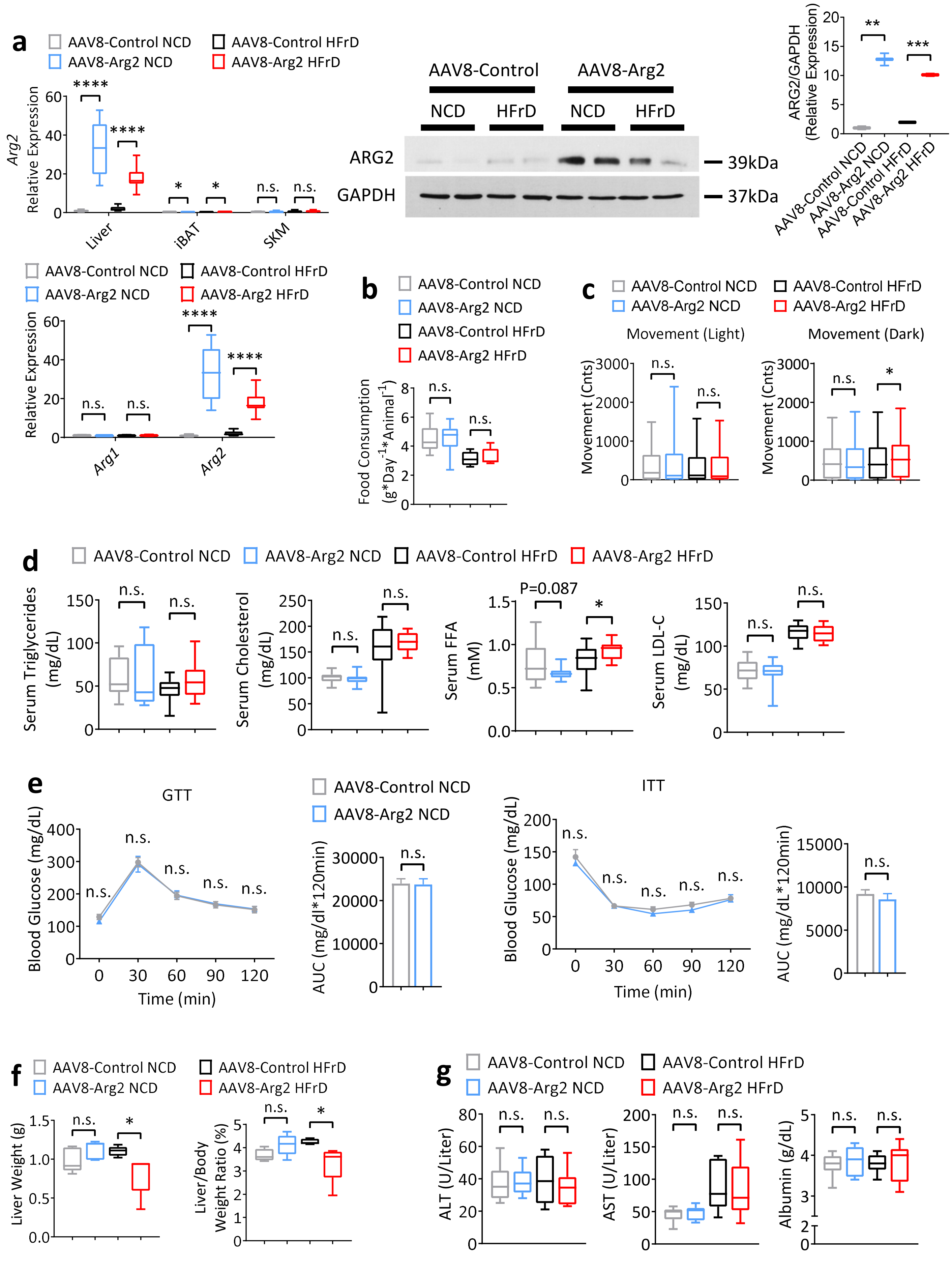

a
AAV8-Control NCD
AAV8-Arg2 NCD
AAV8-Control HFrD
AAV8-Arg2 HFrD
Arg2 Relative Expression
Liver
iBAT
SKM
AAV8-Control
AAV8-Arg2
NCD
HFrD
ARG2
GAPDH
39kDa
37kDa
ARG2/GAPDH (Relative Expression)
Relative Expression
Arg1
Arg2
b
Food Consumption (g*Day-1*Animal-1)
c
Movement (Light)
Movement (Dark)
Movement (Cnts)
d
Serum Triglycerides (mg/dL)
Serum Cholesterol (mg/dL)
Serum FFA (mM)
P=0.087
Serum LDL-C (mg/dL)
e
GTT
Blood Glucose (mg/dL)
Time (min)
AUC (mg/dl*120min)
ITT
AUC (mg/dL*120min)
f
Liver Weight (g)
Liver/Body Weight Ratio (%)
g
ALT (U/Liter)
AST (U/Liter)
Albumin (g/dL)
n.s.

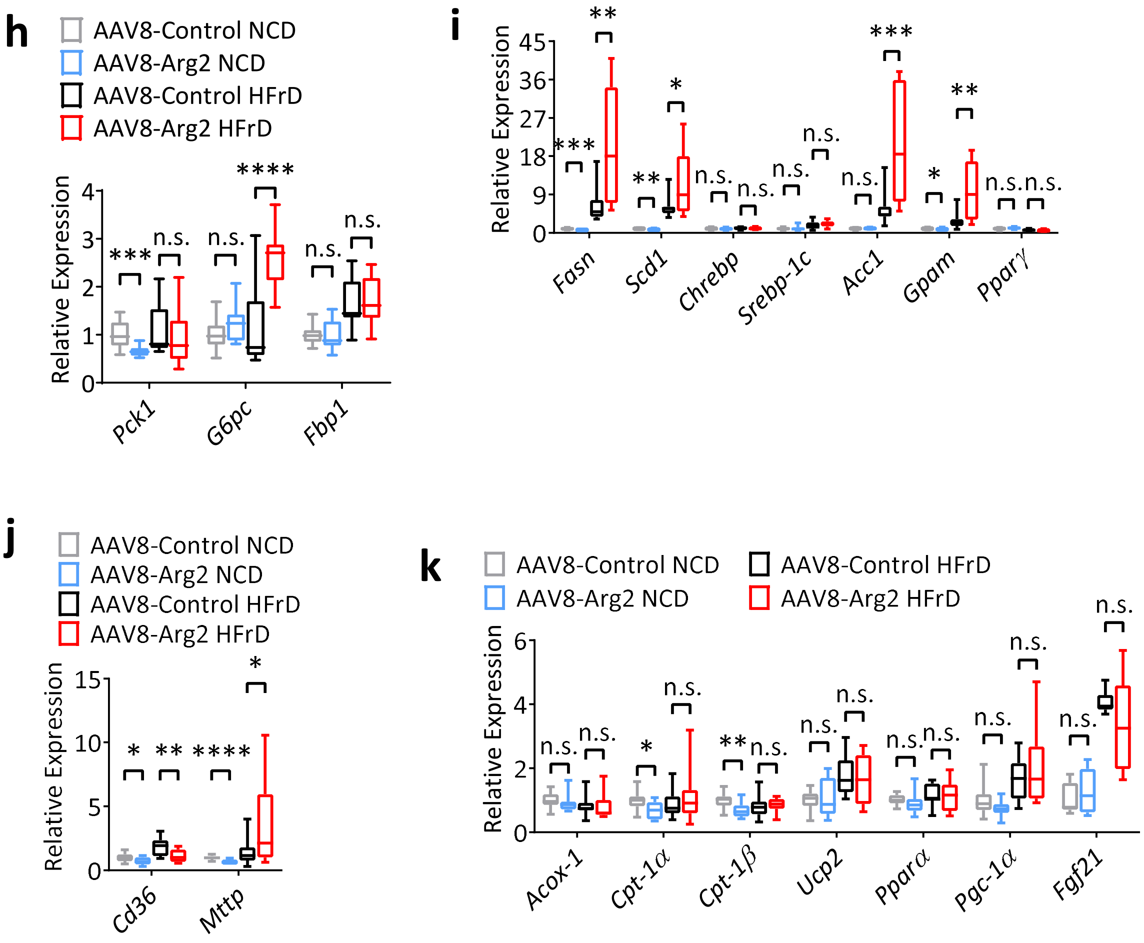

Supplementary Figure 1. Improved energy metabolism in HFrD-fed mice overexpressing hepatocyte Arg2. (**a**) Upper panel: hepatic, interscapular brown adipose tissue (iBAT) and skeletal muscle (SKM) mRNA in AAV8-Control and AAV8-Arg2 mice; Middle panel: hepatic Arg2 protein and densitometric quantification in AAV8-Control and AAV8-Arg2 mice; Lower panel: Hepatic Arg1 and Arg2 expression in AAV8-Control and AAV8-Arg2 mice. (**b**) Food intake in NCD and HFrD-fed mice treated with AAV8-control or AAV8-Arg2 virus. (**c**) Light- and dark-cycle movement. (**d**) Serum triglyceride, cholesterol, non-esterified fatty acid, and LDL-C content in NCD and HFrD-fed mice treated with AAV8-control or AAV8-Arg2 virus. (**e**) Intraperitoneal glucose tolerance test (GTT) and insulin tolerance test (ITT) (n = 5 mice per group). (**f**) Liver weight and liver weight-to-body weight ratios in NCD and HFrD-fed mice treated with AAV8-control or AAV8-Arg2 virus (n = 6-8 mice). (**g**) Serum alanine aminotransferase (ALT), aspartate aminotransferase (AST) and albumin in chow- and HFrD-fed AAV8-Control and AAV8-Arg2 mice. (**h**) Hepatic mRNA expression of gluconeogenic genes. (**I**) Hepatic mRNA expression of genes related in fatty acid synthesis. (**j**) Hepatic mRNA expression of genes related in fatty acid intake and export. (**k**) Hepatic mRNA expression of genes related in fatty acid β-oxidation. (n = 6-8 mice per group). For bar graphs, data represent mean + s.e.m. For box plots, the midline represents the median, boxes represent the interquartile range and whiskers show the full range of values. **P* < 0.05, ***P* < 0.01, ****P* < 0.005, *****P* < 0.0001 relative to vehicle treatment, by two-tailed Student's I-test.

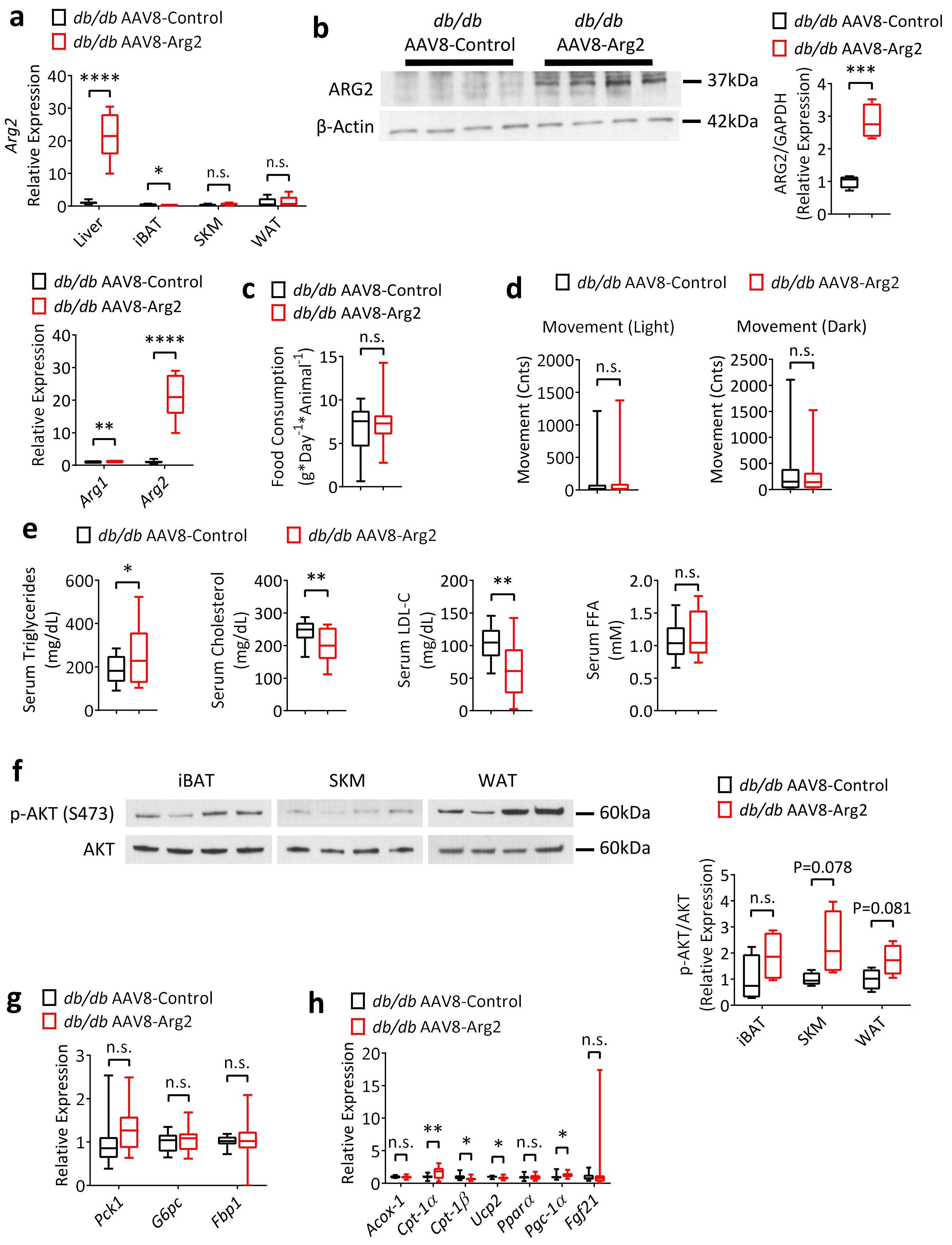

a
db/db AAV8-Control
db/db AAV8-Arg2
Arg2 Relative Expression
****
*
n.s.
n.s.
Liver
iBAT
SKM
WAT
b
db/db AAV8-Control
db/db AAV8-Arg2
ARG2
β-Actin
37kDa
42kDa
db/db AAV8-Control
db/db AAV8-Arg2
***
ARG2/GAPDH (Relative Expression)
db/db AAV8-Control
db/db AAV8-Arg2
Relative Expression
**
****
Arg1
Arg2
c
db/db AAV8-Control
db/db AAV8-Arg2
n.s.
Food Consumption (g*Day^{-1}*Animal^{-1})
d
db/db AAV8-Control
db/db AAV8-Arg2
Movement (Light)
Movement (Dark)
Movement (Cnts)
n.s.
n.s.
e
db/db AAV8-Control
db/db AAV8-Arg2
Serum Triglycerides (mg/dL)
*
Serum Cholesterol (mg/dL)
**
Serum LDL-C (mg/dL)
**
Serum FFA (mM)
n.s.
f
iBAT
SKM
WAT
p-AKT (S473)
AKT
60kDa
60kDa
db/db AAV8-Control
db/db AAV8-Arg2
p-AKT/AKT (Relative Expression)
n.s.
P=0.078
P=0.081
iBAT
SKM
WAT
g
db/db AAV8-Control
db/db AAV8-Arg2
Relative Expression
n.s.
n.s.
n.s.
Pck1
G6pc
Fbp1
h
db/db AAV8-Control
db/db AAV8-Arg2
Relative Expression
n.s.
**
*
*
n.s.
*
n.s.
Acox-1
Cpt-1α
Cpt-1β
Ucp2
Pparα
Pgc-1α
Fgf21

Supplementary Figure 2. Improved energy metabolism in *db/db* mice overexpressing hepatocyte Arg2. (**a**) Left, Hepatic and extrahepatic Arg2 mRNA expression in *db/db* AAV8-Control and AAV8-Arg2 mice; Right, Hepatic Arg1 and Arg2 mRNA expression in *db/db* AAV8-Control and AAV8-Arg2 mice (n = 8 mice per group). (**b**) Arg2 immunoblot analysis and densitometric quantification of immunoblots in livers of *db/db* AAV8-Control and AAV8-Arg2 mice (n = 4 per group). β-actin was probed as a loading control. (**c**) Food consumption in *db/db* AAV8-Control and AAV8-Arg2 mice. (**d**) Light and dark cycle locomotion in *db/db* AAV8-control and Arg2 mice (n = 8 mice per group). (**e**) Serum triglyceride, cholesterol, non-esterified fatty acid, and LDL-C in *db/db* AAV8-Control and AAV8-Arg2 mice (n = 8 mice per group). (**f**) Phosphorylated Akt immunoblot and quantification of immunoblot band density in iBAT, SKM and WAT from *db/db* AAV8-Control or AAV8-Arg2 mice. (**g**) Hepatic gluconeogenic and oxidative gene mRNA expression by qPCR in *db/db* AAV8-Control and AAV8-Arg2 mice (n = 8 mice per group). For boxes represent the interquartile range and whiskers show the full range of values. *$P < 0.05$, **$P < 0.01$, ***$P < 0.005$, ****$P < 0.0001$; n.s., not significant between groups, by two-tailed Student's t-test.

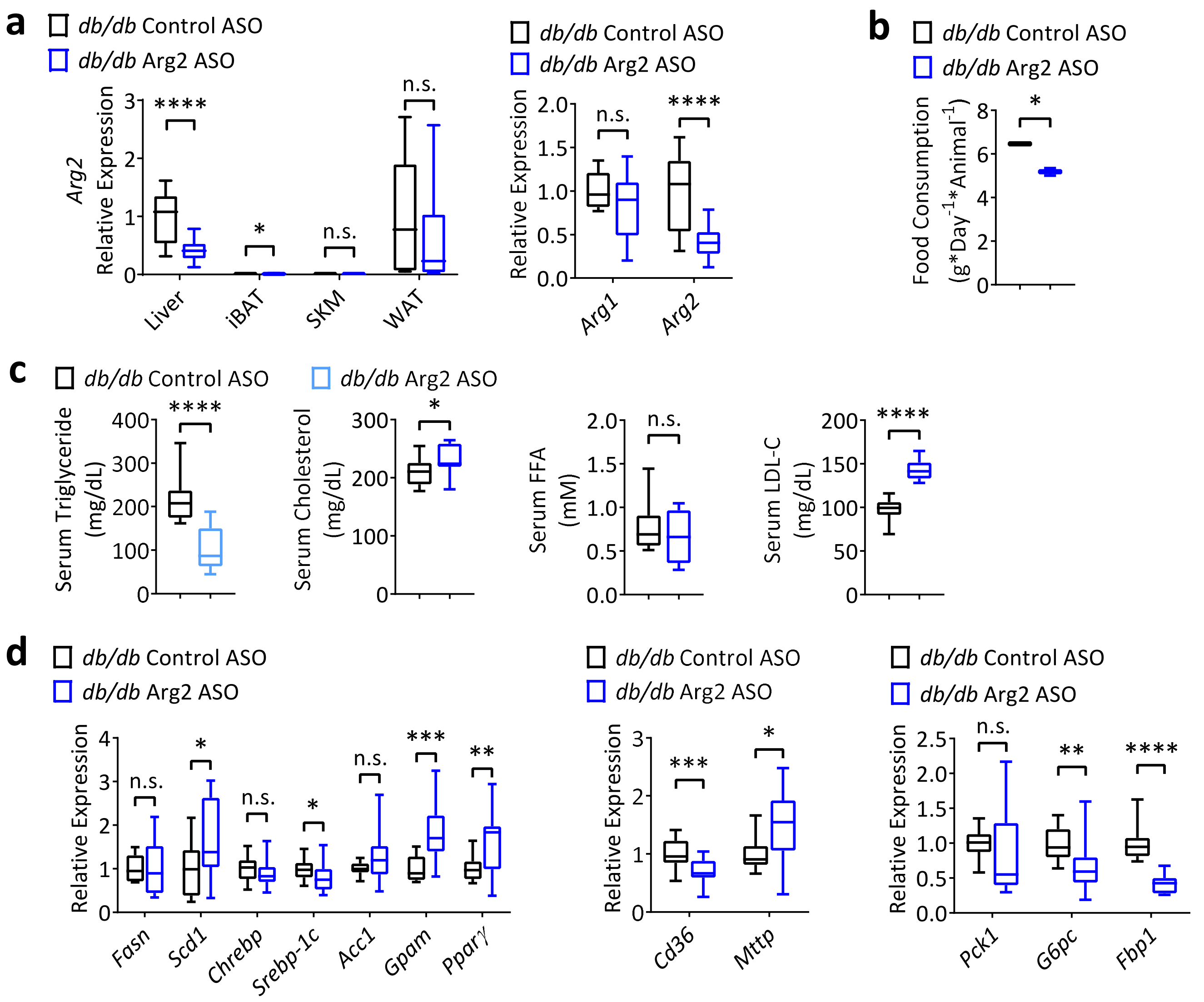

Supplementary Figure 3. Impaired energy metabolism in *db/db* mice treated with Arg2 ASO. (**a**) Left, Hepatic and extrahepatic Arg2 mRNA expression in *db/db* Control and Arg2 ASO mice. Right, Arg1 expression in *db/db* Control and Arg2 ASO mice. (**b**) food consumption in *db/db* Control and Arg2 ASO mice. (**c**) Serum triglyceride, cholesterol, non-esterified fatty acid, and LDL-C in *db/db* Control ASO and Arg2 ASO mice (**d**) Hepatic gluconeogenic, lipid import/efflux and oxidative bar graphs, data represent mean + s.e.m. For box plots, the midline represents the median, gene mRNA expression by qPCR in *db/db* Control ASO and Arg2 ASO mice (n = 8 mice per group). For boxes represent the interquartile range and whiskers show the full range of values. *$P < 0.05$, ****$P < 0.0001$; n.s., not significant between groups, by two-tailed Student's t-test.

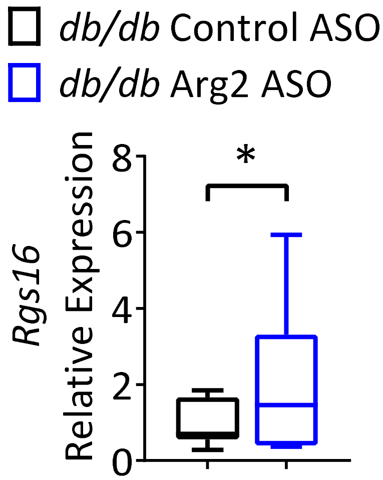

Supplementary Figure 4. RGS16 mRNA expression in livers from *db/db* Control and Arg2 ASO mice. (n = 8 mice per group). For boxes represent the interquartile range and whiskers show the full range of values. *, $P < 0.05$ vs. control, by two-tailed Student's t-test.

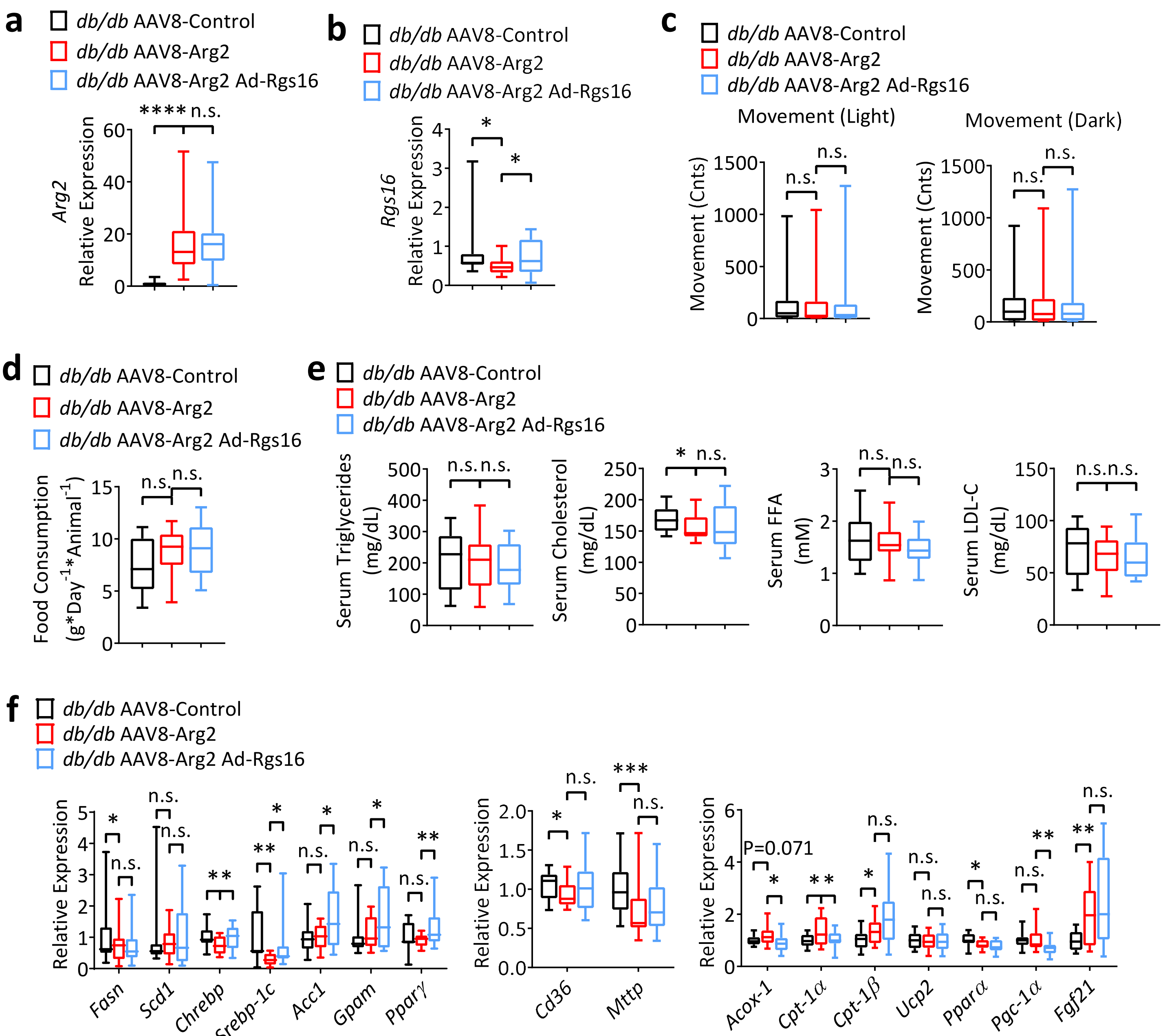

Supplementary Figure 5. Hepatic RGS16 reconstitution impairs energy metabolism in Arg2 overexpressing mice. (**a**) Arg2 and (**b**) RGS16 mRNA expression in *db/db* AAV8-Control or AAV8-Arg2 mice overexpressing control vector or RGS16. (**c**) Light and dark cycle locomotion (**d**) food consumption in *db/db* AAV8-Control or AAV8-Arg2 mice overexpressing control vector or RGS16. (**e**) Serum triglycerides, cholesterol, non-esterified fatty acids and LDL-C in *db/db* AAV8-Control or AAV8-Arg2 mice overexpressing control vector or RGS16. (**f**) Hepatic mRNA gene expression of genes involved in de novo lipogenesis, lipid import/efflux and fatty acid oxidation in *db/db* AAV8-Control or AAV8-Arg2 mice overexpressing control vector or RGS16. For box plots, the midline represents the median, boxes represent the interquartile range and whiskers show the full range of values. *$P < 0.05$, **$P < 0.01$, ***$P < 0.005$, ****$P < 0.0001$; n.s., not significant between groups, by two-tailed Student's t-test.

Figure 1d

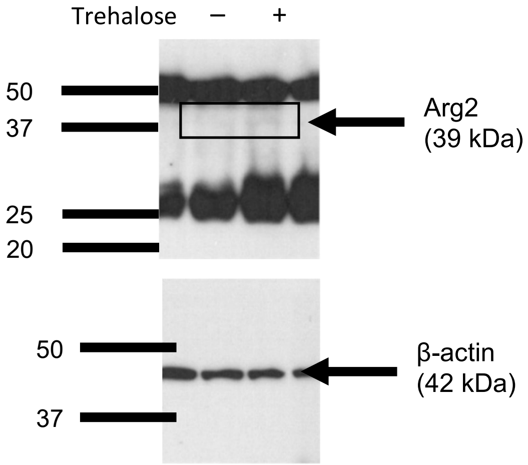

Figure 1e

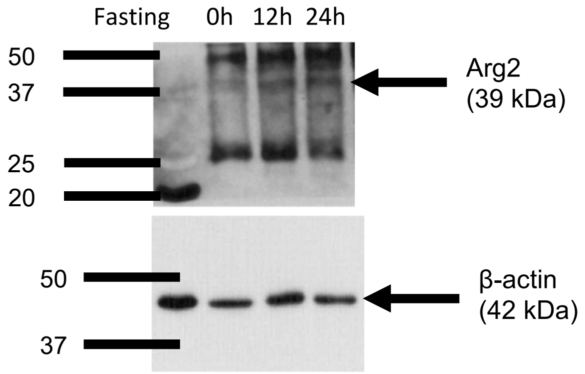

Figure 1h

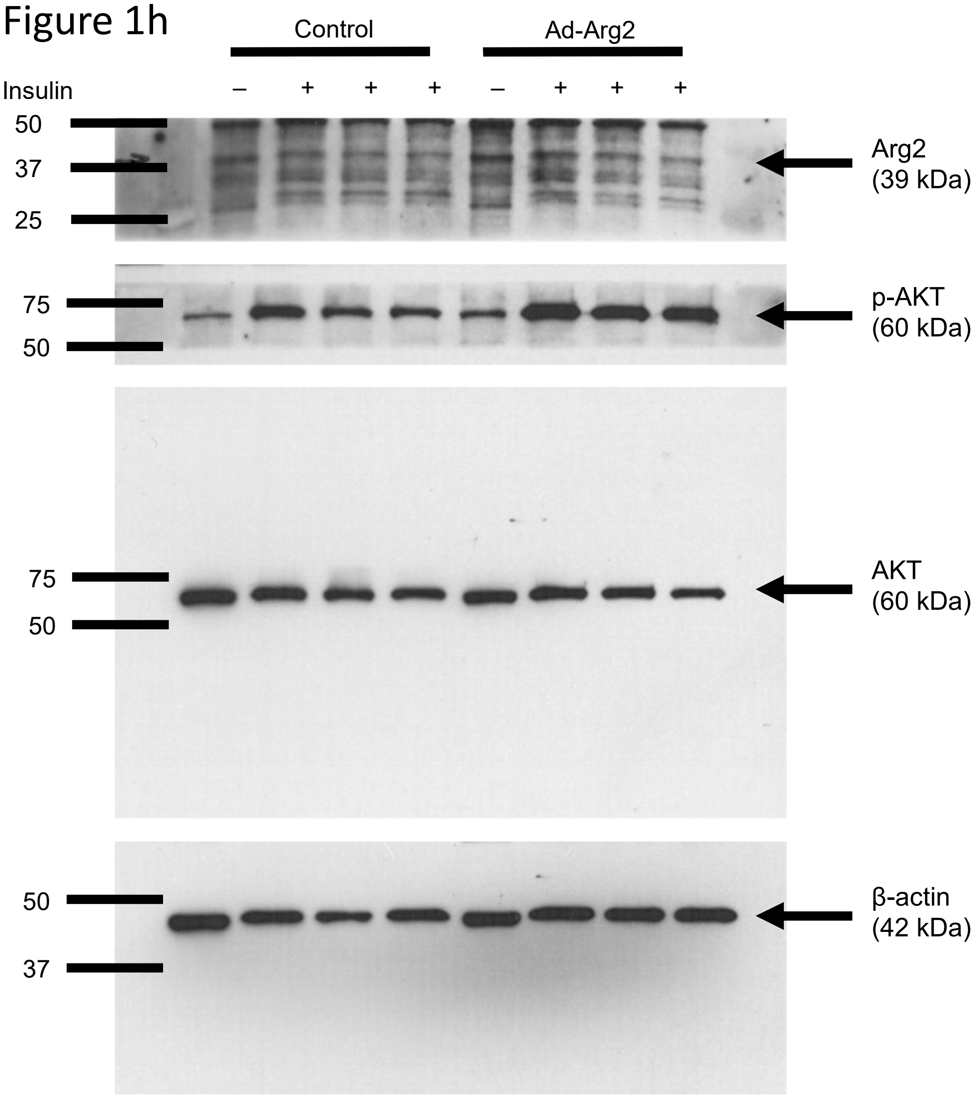

Supplementary Figure 6. The uncropped scans of western blots and gels for Figure 1d, 1e, and 1h.

Figure 4h

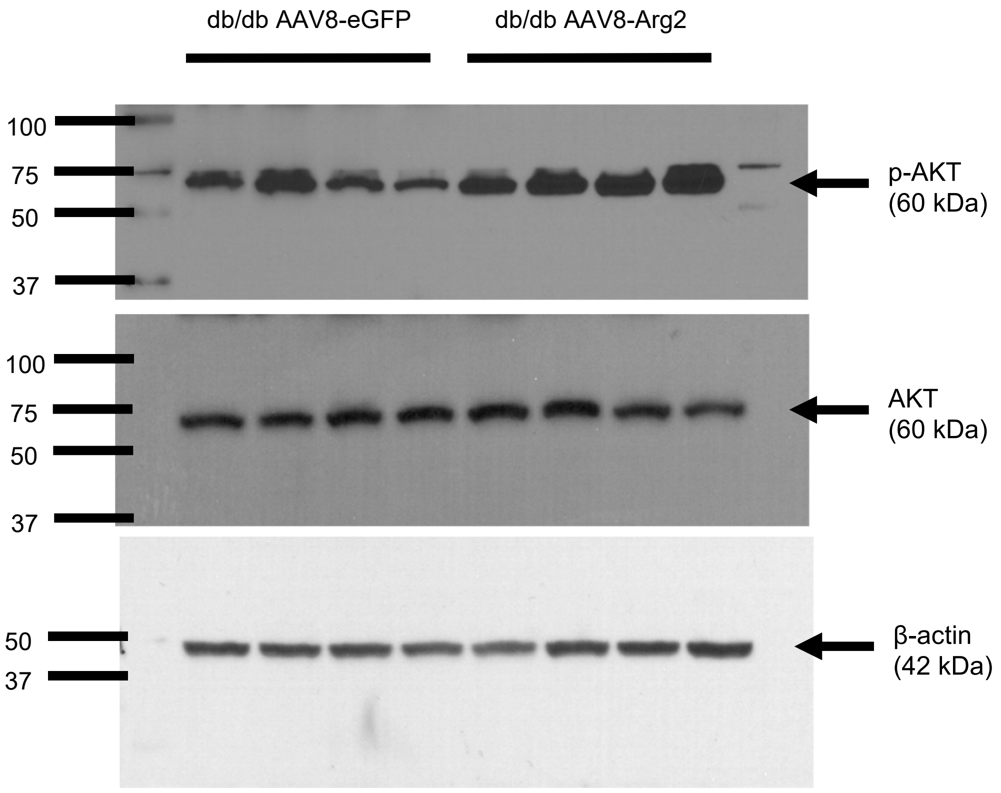

Supplementary Figure 7. The uncropped scans of western blots and gels for Figure 4h.

Figure 8f

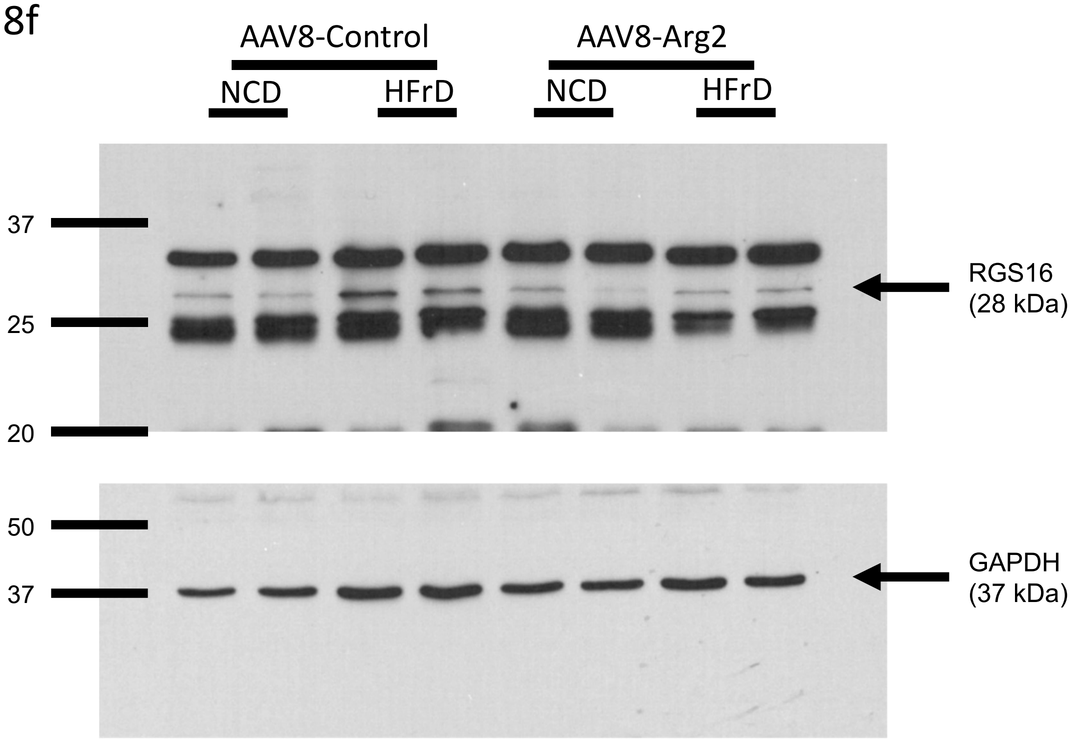

Figure 8g

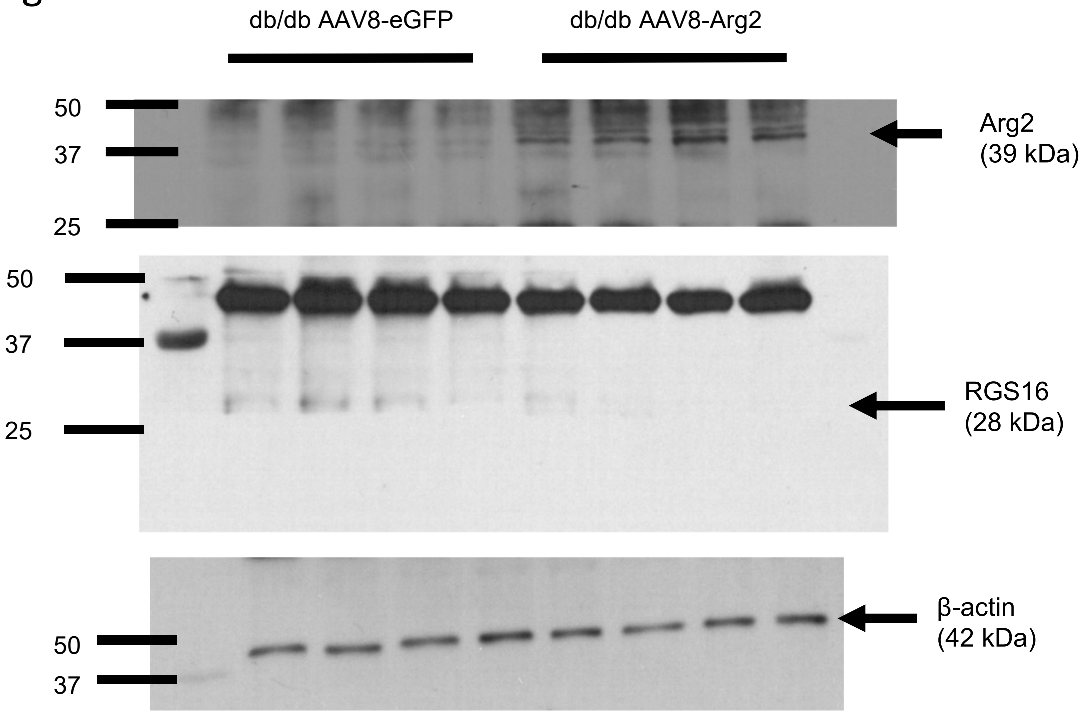

Supplementary Figure 8. The uncropped scans of western blots and gels for Figure 8f and 8g.

Figure 9g

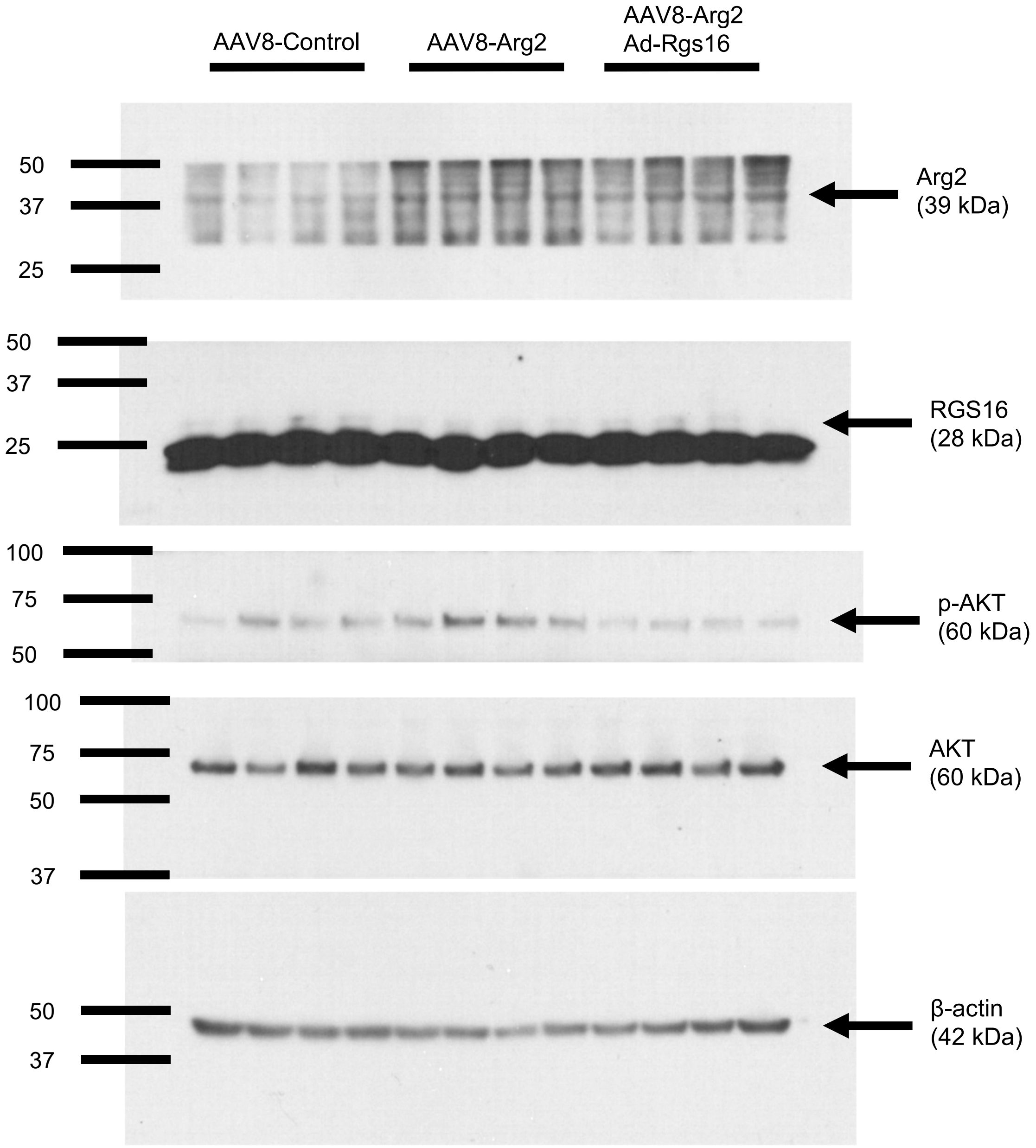

Supplementary Figure 9. The uncropped scans of western blots and gels for Figure 9g.

Supplementary Table 1

	LIVER				SERUM			
Amino Acid	db/db AAV8-Control	db/db AAV8-Arg2	P Value	Corrected P Value	db/db AAV8-Control	db/db AAV8-Arg2	P Value	Corrected P Value
Asp	0.2551 ± 0.013	0.3207 ± 0.0396	0.138	-	0.139 ± 0.01052	0.1795 ± 0.01458 *	0.039	0.8274
**Ala**	**0.8551 ± 0.0415**	**1.131 ± 0.0589 ****	**0.002**	**0.0378***	0.1801 ± 0.007167	0.2444 ± 0.04159	0.127	-
Asn	0.163 ± 0.00566	0.16 ± 0.005645	0.711	-	0.0411 ± 0.002152	0.07538 ± 0.02286	0.133	-
Gly	0.03998 ± 0.002461	0.0761 ± 0.0158 *	0.041	0.8526	0.007464 ± 0.001199	0.0092 ± 0.00258	0.536	-
Gln	0.7092 ± 0.05288	0.5686 ± 0.07607	0.151	-	0.5735 ± 0.03747	0.6194 ± 0.05859	0.510	-
Pro	45.69 ± 1.139	50.35 ± 1.756 *	0.043	0.9051	24.66 ± 2.073	34.87 ± 6.82	0.153	-
Cit	0.3072 ± 0.01812	0.3784 ± 0.03314	0.094	-	2.688 ± 0.1269	2.885 ± 0.241	0.466	-
His	1.43 ± 0.0562	1.574 ± 0.06756	0.125	-	0.494 ± 0.01149	0.5876 ± 0.08059	0.240	-
Arg	0.0729 ± 0.00416	0.1819 ± 0.0370 *	0.013	0.2646	0.0740 ± 0.0096	0.1547 ± 0.0412	0.063	-
Orn	0.311 ± 0.00999	0.3329 ± 0.0182	0.308	-	1.323 ± 0.05483	1.22 ± 0.1443	0.496	-
Lys	3.222 ± 0.127	3.439 ± 0.2011	0.377	-	2.561 ± 0.09872	2.588 ± 0.2243	0.913	-
Trp	0.1406 ± 0.00505	0.1383 ± 0.0044	0.735	-	0.7338 ± 0.04838	0.681 ± 0.01231	0.340	-
Phe	5.068 ± 0.1972	5.022 ± 0.1748	0.865	-	3.178 ± 0.1261	3.6 ± 0.335	0.237	-
Tyr	3.72 ± 0.1219	3.496 ± 0.1963	0.348	-	2.603 ± 0.1608	3.285 ± 0.3652	0.113	-
Leu	2.589 ± 0.1112	2.7 ± 0.1158	0.500	-	2.139 ± 0.1887	2.432 ± 0.2701	0.380	-
Ile	1.832 ± 0.0623	2.053 ± 0.1144	0.112	-	2.362 ± 0.2032	2.931 ± 0.3141	0.143	-
Met	1.725 ± 0.09599	1.898 ± 0.1032	0.239	-	1.516 ± 0.162	2.038 ± 0.2976	0.134	-
Val	1.551 ± 0.04573	1.843 ± 0.1468	0.079	-	2.279 ± 0.1719	2.742 ± 0.2977	0.188	-
Glu	1.373 ± 0.09428	1.766 ± 0.2292	0.135	-	0.4315 ± 0.0388	0.4327 ± 0.0703	0.988	-
Thr	1.611 ± 0.05668	1.498 ± 0.0756	0.251	-	1.142 ± 0.038	1.218 ± 0.09126	0.431	-
Ser	1.632 ± 0.06994	1.725 ± 0.0734	0.370	-	0.7294 ± 0.0304	0.9478 ± 0.1761	0.214	-

Supplementary Table 1. Targeted metabolomic analysis of hepatic extract amino acid and urea cycle intermediaries from *db/db* AAV8-Control and AAV8-Arg2 mice. Data are shown as mean peak height:internal standard ratio (n = 8 mice per group). *, $P < 0.05$.

Supplementary Table 2 qPCR primers

Genes	Forward (5' - 3')	Reverse (5' - 3')
36B4	TAA AGA CTG GAG ACA AGG TG	GTG TAC TCA GTC TCC ACA GA
Acc1	TGT CCG CAC TGA CTG TAA CCA	TGC TCC GCA CAG ATT CTT CA
Acox1	CCT GAT TCA GCA AGG TAG GG	TCG CAG ACC CTG AAG AAA TC
Arg2	AGG AGT GGA ATA TGG TCC AGC	AGG GAT CAT CTT GTG GGA CAT T
Ass1	ACA CCT CCT GCA TCC TCG T	GCT CAC ATC CTC AAT GAA CAC CT
Ccl2	TTA AAA ACC TGG ATC GGA ACC AA	GCA TTA GCT TCA GAT TTA CGG GT
Cd36	GGA ACT GTG GGC TCA TTG C	CAT GAG AAT GCC TCC AAA CAC
Chrebp	CTG GGG ACC TAA ACA GGA GC	GAA GCC ACC CTA TAG CTC CC
Cpt-1α	AGT GGC CTC ACA GAC TCC AG	GCC CAT GTT GTA CAG CTT CC
Cpt-1β	GCA CAC CAG CAG GCA GTA GCT TT	CAG GAG TTG ATT CCA GAC AGG TA
Cxcl9	GGA GTT CGA GGA ACC CTA GTG	GGG ATT TGT AGT GGA TCG TGC
Fasn	CCT GGA TAG CAT TCC GAA CCT	AGC ACA TCT CGA AGG CTA CAC A
Fbp1	CAC CGC GAT CAA AGC CAT CT	AGG TAG CGT AGG ACG ACT TCA
Fgf21	CTG CTG GGG GTC TAC CAA G	CTG CGC CTA CCA CTG TTC C
G6pc	TCT GTC CCG GAT CTA CCT TG	GCT GGC AAA GGG TGT AGT GT
Gck	TGT GGC CAC CGT GTC ATT C	CAA CTG GAC CAA GGG CTT CAA
Gpam	CAA CAC CAT CCC CGA CAT C	GTG ACC TTC GAT TAT GCG ATC A
Igfp1	CCA TCC TGT GGA ACG CCA TC	TCT TGT TGC AGT TTG GCA GAT A
Il-1β	GCA ACT GTT CCT GAA CTC AAC T	ATC TTT TGG GGT CCG TCA ACT
Il-6	CTG CAA GAG ACT TCC ATC CAG	AGT GGT ATA GAC AGG TCT GTT GG
Irs1	CGA TGG CTT CTC AGA CGT G	CAG CCC GCT TGT TGA TGT TG
Mogat1	TGG TGC CAG TTT GGT TCC AG	TGC TCT GAG GTC GGG TTC A
Mttp	ATG ATC CTC TTG GCA GTG CTT	TGA GAG GCC AGT TGT GTG AC
Pck1	GAT GGG CAT ATC TGT GCT GG	CAG CCA CCC TTC CTC CTT AG
Pgc1α	ACA CCG CAA TTC TCC CTT GT	CGG CGC TCT TCA ATT GCT TT
Pparα	TGG TTC CTG GTG CCG ATT TA	ACT AGC ATC CCA CTT AAT TAT GTA TCT
Pparγ	CCA CCA ACT TCG GAA TCA GCT	TTT GTG GAT CCG GCA GTT AAG A
Rgs16	CCA TGC CTT CCT AAA GAC GGA	GTA CTC GTC AAA GAT GTG GTG AG
Srebp-1c	CCA TGG ATT GCA CAT TTG AA	GGC CAG GGA AGT CAC TGT CTT
Tnfα	CAG GCG GTG CCT ATG TCT C	CGA TCA CCC CGA AGT TCA GTA G
Ucp2	ATG GTT GGT TTC AAG GCC ACA	CGG TAT CCA GAG GGA AAG TGA T
