# Supplementary material for: Hepatic arginase 2 (Arg2) is sufficient to convey the therapeutic metabolic effects of fasting
Source: Nat Commun. 2019 Apr 8;10:1587. doi: 10.1038/s41467-019-09642-8 (PMC6453920; doi:10.1038/s41467-019-09642-8)
Supplement: Supplementary file 3 — Reporting Summary [file 41467_2019_9642_MOESM3_ESM.pdf]

nature research

Corresponding author(s): Brian J DeBosch, MD, PhD

Last updated by author(s): Mar 19, 2019

Reporting Summary

Nature Research wishes to improve the reproducibility of the work that we publish. This form provides structure for consistency and transparency in reporting. For further information on Nature Research policies, see Authors & Referees and the Editorial Policy Checklist.

Statistics

For all statistical analyses, confirm that the following items are present in the figure legend, table legend, main text, or Methods section.

n/a	Confirmed	
☐	☒	The exact sample size (*n*) for each experimental group/condition, given as a discrete number and unit of measurement
☐	☒	A statement on whether measurements were taken from distinct samples or whether the same sample was measured repeatedly
☐	☒	The statistical test(s) used AND whether they are one- or two-sided<br>*Only common tests should be described solely by name; describe more complex techniques in the Methods section.*
☐	☒	A description of all covariates tested
☐	☒	A description of any assumptions or corrections, such as tests of normality and adjustment for multiple comparisons
☐	☒	A full description of the statistical parameters including central tendency (e.g. means) or other basic estimates (e.g. regression coefficient) AND variation (e.g. standard deviation) or associated estimates of uncertainty (e.g. confidence intervals)
☐	☒	For null hypothesis testing, the test statistic (e.g. *F*, *t*, *r*) with confidence intervals, effect sizes, degrees of freedom and *P* value noted<br>*Give P values as exact values whenever suitable.*
☒	☐	For Bayesian analysis, information on the choice of priors and Markov chain Monte Carlo settings
☐	☒	For hierarchical and complex designs, identification of the appropriate level for tests and full reporting of outcomes
☒	☐	Estimates of effect sizes (e.g. Cohen's *d*, Pearson's *r*), indicating how they were calculated

Our web collection on statistics for biologists contains articles on many of the points above.

Software and code

Policy information about availability of computer code

Data collection	No software was used for data collection.
Data analysis	Prism version 6.0 and 7.0 (GraphPad Software, San Diego, CA).<br>Past 3.20 (Natural History Museum, University of Oslo, Norway).

For manuscripts utilizing custom algorithms or software that are central to the research but not yet described in published literature, software must be made available to editors/reviewers. We strongly encourage code deposition in a community repository (e.g. GitHub). See the Nature Research guidelines for submitting code & software for further information.

Data

Policy information about availability of data

All manuscripts must include a data availability statement. This statement should provide the following information, where applicable:

- Accession codes, unique identifiers, or web links for publicly available datasets
- A list of figures that have associated raw data
- A description of any restrictions on data availability

The authors declare that [the/all other] data supporting the findings of this study are available within the paper [and its supplementary information files]. Raw RNA sequencing data files have been deposited into the Gene Expression Omnibus data-base (www.ncbi.nlm.nih.gov/geo/) with accession number GSE126134.

Field-specific reporting

Please select the one below that is the best fit for your research. If you are not sure, read the appropriate sections before making your selection.

☒ Life sciences ☐ Behavioural & social sciences ☐ Ecological, evolutionary & environmental sciences

For a reference copy of the document with all sections, see nature.com/documents/nr-reporting-summary-flat.pdf

Life sciences study design

All studies must disclose on these points even when the disclosure is negative.

Sample size	We performed pilot studies with n = 4 per treatment group. We found these sample sizes to be sufficient to detect differences in our primary outcome (hepatic triglyceride content). We then repeated animal cohorts using similar or larger sample sizes to validate our pilot findings.
Data exclusions	Figs 3 and 4 represent combined data from two separate cohorts of db/db mice treated with or without AAV8-Arg2. Four db/db mice (two excluded from AAV8 Controls and two excluded from AAV8 Arg2 groups) were excluded from the analysis. Two were of normal body weight and two were excluded because of weight loss (20-25% of peak body weight) and ill appearance at the end of the study. We pre-determined that any signs of illness, (e.g. weight loss, failed grooming, hunched/fluffed appearance, etc.), would preclude analysis of any given animal.
Replication	All attempts at replication were successful.
Randomization	All mice were randomly assigned to experimental groups.
Blinding	Investigators were not blinded during animal experiments.

Reporting for specific materials, systems and methods

We require information from authors about some types of materials, experimental systems and methods used in many studies. Here, indicate whether each material, system or method listed is relevant to your study. If you are not sure if a list item applies to your research, read the appropriate section before selecting a response.

Materials & experimental systems

n/a	Involved in the study
☐	☒ Antibodies
☐	☒ Eukaryotic cell lines
☒	☐ Palaeontology
☐	☒ Animals and other organisms
☒	☐ Human research participants
☒	☐ Clinical data

Methods

n/a	Involved in the study
☒	☐ ChIP-seq
☒	☐ Flow cytometry
☒	☐ MRI-based neuroimaging

Antibodies

Antibodies used	Antibodies against phosphor-IRS1 (Ser636/639) (no. 2388), IRS1 (no. 2382), phosphor-AKT (Ser473) (no. 9271), AKT (no. 9272), GAPDH (no. 5174), and β-actin (no. 3700) were pur-chased from Cell Signaling Technology (CST) (Beverly, MA, USA). Anti-Arg2 antibody was pur-chased from Santa Cruz Biotechnology (no. Ab154422). Antibody against RGS16 (no. NBP2-01584) was purchased from Novus Biologicals, LLC (no. Ab203071) Littleton, CO, USA). The dilution ratio for all primary antibodies was 1:1,000. The secondary antibodies used in this study were peroxidase-conjugated anti—rabbit IgG (A7074) and anti—mouse IgG (A7076) purchased from CST, in which were used at a 1:5,000 dilution.
Validation	The antibodies have been either validated in previous studies or validated with positive controls in the current study.

Eukaryotic cell lines

Policy information about cell lines

Cell line source(s)	Primary hepatocytes, isolated from male C57B/6 mice. AML12 mouse cell lines (obtained from ATCC directly).
Authentication	AML12 cell lines were purchased directly from ATCC and propagated in our laboratory. These cell lines were authenticated at the ATCC.
Mycoplasma contamination	We did not test for mycoplasma.

Commonly misidentified lines (See ICLAC register)	No commonly misidentified lines were used in this current study.

Animals and other organisms

Policy information about studies involving animals; ARRIVE guidelines recommended for reporting animal research

Laboratory animals	C57B/6 males obtained directly from Jackson Laboratory, age 5-11wks of age were used in all studies, both for db/db studies and high-fructose fed studies.
Wild animals	The study did not involve wild animals.
Field-collected samples	The study did not involve samples collected from the field.
Ethics oversight	All animals received humane care and procedures were performed in accordance with the ap-proved guidelines by the Animal Studies Committee at Washington University School of Medi-cine. All animal studies were performed in accordance with the criteria and ethical regulations outlined by the Institutional Animal Care and Use Committee (IACUC).

Note that full information on the approval of the study protocol must also be provided in the manuscript.
